# A simple technique to identify key recruitment issues in randomised controlled trials: Q-QAT - quanti-qualitative appointment timing

**DOI:** 10.1186/1745-6215-14-S1-O62

**Published:** 2013-11-29

**Authors:** Sangeetha Paramasivan, Sean Strong, Caroline Wilson, Jane Blazeby, Jenny Donovan

**Affiliations:** 1 School of Social and Community Medicine, University of Bristol, Bristol, UK

## Background

Recruitment to pragmatic randomised controlled trials (RCTs) is acknowledged to be difficult, but few interventions to improve recruitment have proved to be effective. We present a simple technique aimed at improving recruitment to RCTs by employing mixed research methods to identify key recruitment issues.

## Method

A programme of qualitative research investigating the process of recruitment in six RCTs with recruitment difficulties consistently revealed imbalances in the presentation of treatment arms by clinicians. The Q-QAT technique was developed to quantify this imbalance and involved coding of audio-recorded recruitment appointment transcripts for time spent explaining each of the RCT arms and the RCT itself. The technique was applied in two RCTs with different clinical contexts, organisational issues and recruitment processes. Comparisons of Q-QAT data were made across clinical centres, recruiters and specialties. Findings were fed-back to recruiters and recruitment rates re-assessed.

## Results

In both RCTs there were considerable variations in the quantity of time spent by different recruiters on the RCT arms and the RCT itself. In RCT1, Q-QAT data showed that similar proportions of time were spent explaining each treatment arm, but very little time was spent discussing the RCT itself. In RCT2, treatments were presented very differently across specialties and centres, with specialists spending more time on and recommending their own treatments. Findings were discussed with recruiters. Subsequent changes in Q-QAT patterns, information presentation and recruitment rates were observed.

## Conclusion

The Q-QAT method is easy to apply and rapidly offers opportunities to encourage improvement in recruitment practice.

